# Supplementary material for: A high-resolution mRNA expression time course of embryonic development in zebrafish
Source: eLife. 2017 Nov 16;6:e30860. doi: 10.7554/eLife.30860 (PMC5690287; doi:10.7554/eLife.30860)
Supplement: Supplementary file 6. [file elife-30860-supp6.zip › biolayout-clusters-files/Cluster199-genes.html]

Cluster199


# Cluster199: Genes

| Ensembl ID | Gene Name | Chr | Start | End | Biotype |
| --- | --- | --- | --- | --- | --- |
| ENSDARG00000039669 | TSTA3 (1 of many) | 20 | 52686576 | 52720064 | protein\_coding |
| ENSDARG00000002336 | dlc | 15 | 20527716 | 20532570 | protein\_coding |
| ENSDARG00000000943 | dzip1 | 6 | 7092394 | 7123130 | protein\_coding |
| ENSDARG00000078561 | lrig2 | 6 | 47810996 | 47841706 | protein\_coding |
| ENSDARG00000033537 | p4ha1a | 12 | 9504281 | 9547827 | protein\_coding |
| ENSDARG00000059333 | si:ch211-285f17.1 | 24 | 9231575 | 9456201 | protein\_coding |
| ENSDARG00000079255 | zgc:174935 | 16 | 17804511 | 17807155 | protein\_coding |
